# Multifunctional bilayer scaffold for dental pulp protection and sustained calcium hydroxide release for mineralized tissue regeneration

**DOI:** 10.1016/j.biomaterials.2025.123700

**Published:** 2025-09-16

**Authors:** Caroline Anselmi, Igor P. Mendes Soares, Renan Dal-Fabbro, Sarah Chang, Ana Beatriz Gomes de Carvalho, Pedro H.C. Oliveira, Alexandre H. dos Reis-Prado, Carlos A. de Souza Costa, Josimeri Hebling, Marco C. Bottino

**Affiliations:** a Department of Cardiology, Restorative Sciences, and Endodontics, School of Dentistry, University of Michigan, Ann Arbor, MI, USA; b Department of Morphology and Pediatric Dentistry, São Paulo State University (UNESP), School of Dentistry, Araraquara, Brazil; c Department of Dental Materials and Prosthodontics, São Paulo State University (UNESP), School of Dentistry, Araraquara, Brazil; d Department of Dental Materials and Prosthodontics, São Paulo State University (UNESP), School of Dentistry, São José dos Campos, Brazil; e Department of Preventive and Restorative Dentistry, São Paulo State University (UNESP), School of Dentistry, Araçatuba, Brazil; f Department of Restorative Dentistry, Minas Gerais Federal University (UFMG), School of Dentistry, Belo Horizonte, Minas Gerais, Brazil; g Department of Physiology and Pathology, São Paulo State University (UNESP), School of Dentistry, Araraquara, Brazil; h Department of Biomedical Engineering, College of Engineering, University of Michigan, Ann Arbor, MI, USA

**Keywords:** Polyethylene oxide–polycaprolactone, copolymer, Polycaprolactone, Dental pulp, Guided tissue regeneration, Calcium hydroxide, Dental pulp capping

## Abstract

Injury to the mineralized tissues that protect the dental pulp can lead to pulp exposure and inflammation, necessitating the use of biologically active materials capable of preserving pulp vitality. While current biomaterials such as mineral trioxide aggregate (MTA) are widely used in vital pulp therapy (VPT), their high cost, challenging handling, and lack of structural flexibility highlight the need for alternatives. Here, we introduce a multifunctional bilayer scaffold composed of a polycaprolactone (PCL) film and a PCL/poly(ethylene oxide) (PCL/PEO) blend loaded with calcium hydroxide (CH), designed to provide both cytoprotection and mineralized tissue regeneration. The scaffold features a compact PCL layer, acting as a barrier to protect the pulp from external cytotoxic agents, and a CH-loaded fibrillar PCL/PEO electrospun layer, aimed at promoting odontoblastic differentiation through sustained calcium ion release. The bilayer structure demonstrated mechanical stability and a degradation profile suitable for clinical application. The release mechanism relies on gradual fiber degradation and CH dissolution. *In vitro*, the fibrillar layer enhanced calcium ion release, supported dental pulp stem cell adhesion and viability, and stimulated mineralized matrix formation. The compact layer preserved cell viability even in the presence of glass ionomer cement. *In vivo*, the bilayer scaffold elicited a comparable inflammatory response and expression of dentinogenesis and angiogenesis markers relative to MTA, although it did not attain the same level of mineralized tissue formation. Overall, our results indicate that this multifunctional bilayer scaffold offers a cost-effective, dual-purpose alternative to current materials, with potential for further optimization of its tissue regeneration capabilities prior to clinical implementation.

## 1. Introduction

Preserving pulp vitality remains a central goal of conservative treatments like vital pulp therapy (VPT), which includes pulp capping and pulpotomy [1]. These methods aim to regenerate dentin and preserve the pulp’s protective and regenerative functions [1]. Successful VPT outcomes depend on biomaterials that meet specific clinical and biological standards, such as biocompatibility, bioactivity, antibacterial and anti-inflammatory properties, mechanical stability, radiopacity, and sealing ability [1,2]. Calcium hydroxide (CH) has long been used for its bioactivity and antibacterial effects, but its high solubility, weak mechanical strength, and vulnerability to bacterial microleakage are significant drawbacks [3,4]. Mineral trioxide aggregate (MTA) has become a more reliable alternative, showing improved outcomes in pulp healing and dentin bridge formation. However, it also faces issues like high cost and handling difficulties [4]. Notably, many of MTA’s biological effects are linked to the sustained release of CH during hydration, which contributes to its alkaline pH and calcium ion release [5]. Therefore, directly incorporating CH into a delivery platform offers a logical approach to harnessing its bioactivity while overcoming MTA’s limitations through a tunable and accessible scaffold-based therapy.

Tissue engineering principles based on the cell-homing process aim to stimulate the host tissue’s natural regenerative abilities [6]. This approach is represented by the well-established tissue engineering triad, which includes a support scaffold, signaling molecules, and a cell population. In this model, scaffolds combined with signaling molecules provide temporary support and critical biochemical cues, allowing undifferentiated cells within the dental pulp to migrate, proliferate, and differentiate. Once these cells assume an odontoblastic phenotype, they can secrete matrix proteins to promote dentin formation and seal the pulp exposure [7,8]. In the context of regenerative strategies for VPT, scaffold characteristics such as porosity, interconnectivity, and hydrophilicity are essential to support stem cell adhesion and enable effective nutrient and waste exchange [9]. However, these features also influence scaffold permeability and the potential diffusion of cytotoxic byproducts from restorative materials toward the pulp. To address this, a bilayer scaffold design may provide a practical solution: the layer adjacent to the restoration could be dense and impermeable to ensure sealing and mechanical protection, while the layer in contact with the pulp could be porous, hydrophilic, and designed for controlled release of therapeutic compounds to promote mineralized tissue formation. Moreover, to provide adequate support, the scaffold layer in contact with the pulp should have a structure that can mimic the fibrillar nature of the extracellular matrix (ECM), creating a suitable microenvironment for cell adhesion, proliferation, and differentiation [10].

The electrospinning technique allows the processing of natural and synthetic polymers into fibrous scaffolds that replicate the native extracellular matrix (ECM) [11,12]. This method is regarded as simple and versatile because it enables the achievement of different fiber diameters and pore sizes by altering the applied parameters [12]. Additionally, scaffolds must exhibit a degradation rate that aligns with neotissue formation, being gradually replaced by the newly formed tissue. Polymer blends have been utilized in tissue engineering to manage the scaffold’s degradation profile [13–16]. Polycaprolactone (PCL), a synthetic polymer extensively used for its favorable applicability and processability, demonstrates hydrophobic behavior and a slow degradation rate [17–19]. PCL electrospun fibers have been reported for dentin-pulp complex regeneration, illustrating the upregulation of dentinogenesis-related genes when combined with specific biological cues [20–22]. Importantly, to align with the target tissue maturation rate, the wettability and degradation speed of PCL can be adjusted by blending it with an amphiphilic polymer such as poly(ethylene oxide) (PEO), resulting in a material with enhanced hydrophilicity [14,16].

Despite its proven biocompatibility, PCL requires suitable biological signaling to promote osteo/odontogenic phenotype expression in pulp cells [17,18,23,24]. Therefore, incorporating bioactive compounds into scaffolds, especially by adding calcium phosphate-based inorganic particles, has demonstrated the ability to create a microenvironment that fosters osteo/odontogenic differentiation, resulting in tissue matrix deposition and mineralization [20–22,25–27]. Integrating calcium hydroxide (CH) particles into PCL/PEO fibers could improve the bioavailability of Ca^2+^ ions in the extracellular environment, providing bioactivity to the fibrillar layer and encouraging pulp cells to produce and mineralize the dentin matrix. Recent studies have successfully integrated CH into PCL nanofibrous scaffolds, resulting in increased expression of odontogenic genes by human dental pulp cells (HDPCs) [20–22,26,28,29].

Here, we introduce a bilayer scaffold that addresses two key challenges in VPT. We designed a compact layer to create a reliable seal over exposed pulp tissue, preventing the diffusion of toxic components from cavity liner (restorative) materials. Simultaneously, a fibrillar layer was engineered to support the differentiation of pulp cells into an odontoblastic phenotype, aiming to promote mineralized tissue formation through sustained calcium hydroxide release. This dual-function bilayer scaffold holds clinical promise as it effectively protects the pulp against potentially toxic material components and supports mineralized tissue regeneration in a clinically relevant scenario of vital pulp therapy.

## 2. Results and discussion

### 2.1 Synthesis and characterization of the compact layer

**Fig. 1.**
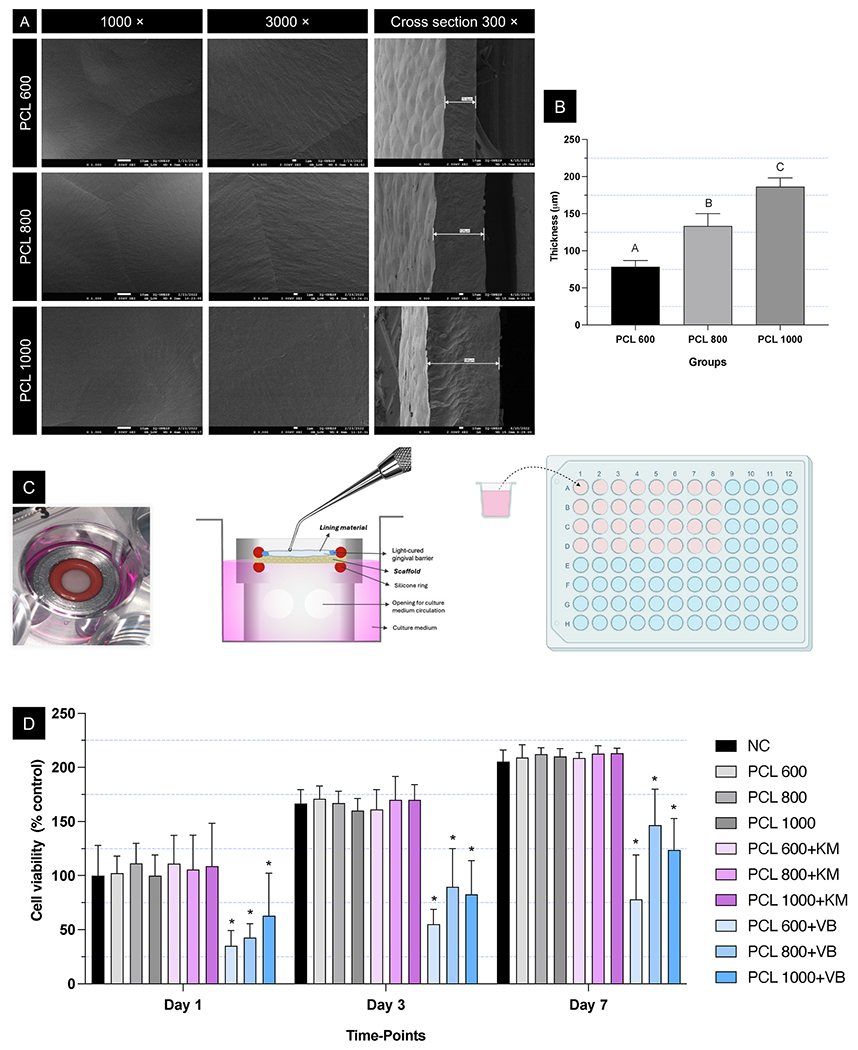
Compact layer morphological characterization and cytocompatibility when combined with glass ionomer cements. **(A)** Representative SEM images of the surface and cross-section of the compact layers. **(B)** Thickness values of the compact layers. Mean and standard deviations. Different letters indicate significant differences between groups (ANOVA/Tukey; n = 12; α = 5 %). **(C)** From left to right: artificial pulp chamber (APC) set up with the compact layer placed in a 24-well culture plate compartment filled with culture medium. Longitudinal section diagram of the APC inserted into the culture plate and application of the glass-ionomer cement on the surface of the compact layer; Application of the collected extracts on hDPSCs pre-seeded in 96-well plates. **(D)** Viability of hDPSCs after 24h of exposure to extracts collected from glass ionomer cements (KM or VB) applied on the different compact layer formulations (600, 800, and 1000). Values calculated considering the negative control (NC) group at day 1 as 100 %. Asterisks (*) indicate significant differences compared to NC (Dunnett’s multiple Comparison Test for each time-point; n =8; α =5 %). Abbreviations: PCL, polycaprolactone; KM, Ketac Molar Easymix; VB, Vitrebond.

Different PCL volumes (PCL 600, 800, and 1000) were used to obtain compact layers of varying thickness to examine their cytoprotection role when combined with distinct glass ionomer cements (GICs). SEM analysis showed a uniform polymeric surface devoid of pores for all three polymer volumes (Fig. 1a). As expected, the thickness of the compact layer increased with the volume of polymer added (p < 0.0001; Fig. 1b). However, this variation in thickness did not affect the sealing ability of the compact layer when combined with either GIC. For the conventional GIC (*i.e.*, Ketac Molar Easymix), all tested thicknesses (ranging from ~75 to 180 μm) prevented cytotoxic effects, indicating effective sealing even at the lowest thickness (p ≥ 0.1090; Fig. 1c and d). In contrast, when a resin-modified GIC (*i.e.*, Vitrebond) was applied, cytotoxicity was observed at all thicknesses, with no significant difference in intensity across the range tested. These results suggest that while the compact PCL layer effectively hampers diffusion of cytotoxic components from conventional GIC, it is less effective against the more permeable and reactive components present in resin-modified GICs. The cytotoxicity of Vitrebond has been linked to the diffusion of 2-hydroxyethyl methacrylate (HEMA) [30], which may not have been completely blocked by the compact layer. Compared to conventional GIC, Vitrebond has a lower powder-liquid ratio (1.4:1.0 versus 4.5:1.0), contributing to its higher fluidity. Additionally, HEMA is known to interact with PCL, forming amphiphilic copolymers that could compromise the barrier properties of the compact layer [31,32]. As a result, the unreacted HEMA may have diffused into the culture medium, reducing cell viability due to its toxicity to pulp cells [33,34]. Therefore, the protective role of the PCL barrier depends not only on its thickness but also on the chemical nature of the overlying material.

### 2.2. Screening of the PCL/PEO polymer ratio of the fibrillar layer

**Fig. 2.**
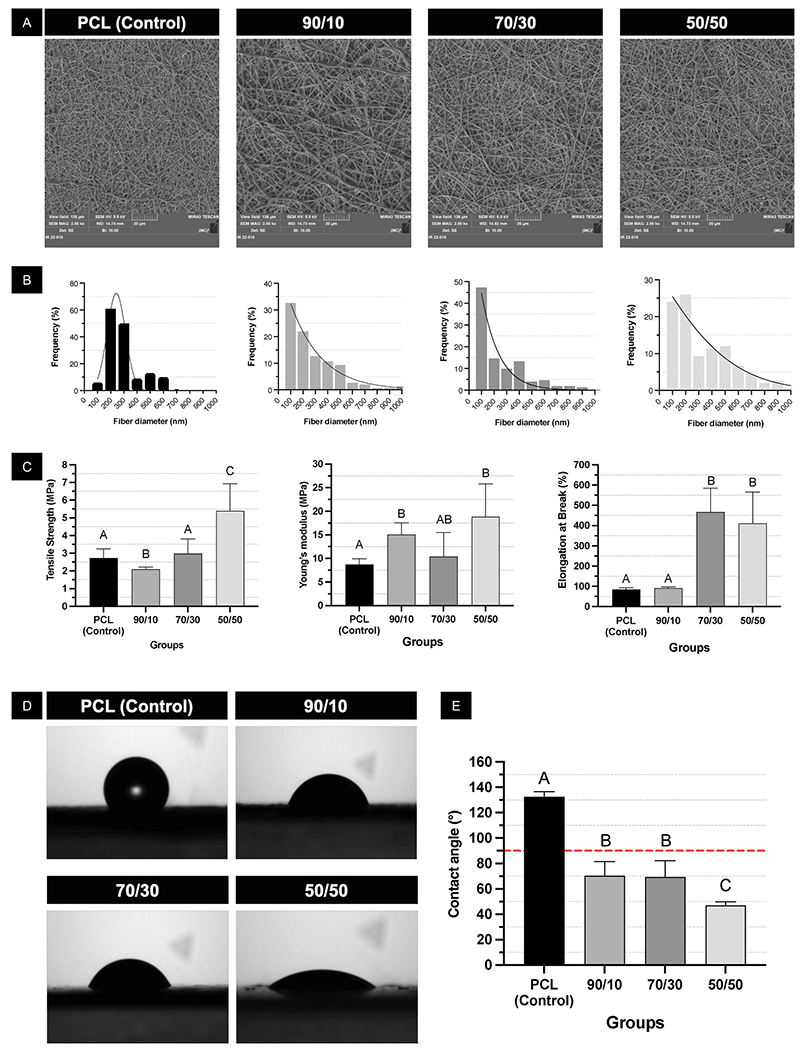
Morphological and mechanical characterization of the PCL/PEO fibrillar layer. **(A)** Representative SEM images of the surface of the fibrillar scaffolds (5000 ×) **(B)** Fiber diameter-frequency plots of the scaffolds (n =150). **(C)** Graphs displaying the mechanical properties of the experimental scaffolds. Mean and standard deviations. Different letters denote statistically significant difference (ANOVA and Welch’s ANOVA/Tukey and Games-Howell; n = 6; α = 5 %). **(D)** Images of a 1 μL water droplet on the surface of the scaffolds. **(E)** Bar graphs representing the contact angle between the ultrapure water droplet and the surface of the scaffolds. Mean and standard deviation. Different letters indicate statistically significant differences between groups (ANOVA/Tukey; n = 6; α = 5 %). The horizontal red dashed line indicates the 90°limiting contact angle between the hydrophobic and hydrophilic properties. (For interpretation of the references to color in this figure legend, the reader is referred to the Web version of this article.)

To optimize fiber formation, different PCL/PEO ratios (100/0, 90/10, 70/30, and 50/50) were tested for their ability to produce uniform and reproducible fibrous meshes through electrospinning. All tested ratios successfully created a uniform fibrillar mesh with randomly oriented fibers (Fig. 2a). The fiber diameter distribution showed a similar pattern, with most fibers measuring between 100 and 200 nm, regardless of the PCL/PEO ratio. However, a higher PEO content led to a broader range of fiber diameters (Fig. 2b). All formulations formed interconnected fiber networks, which are essential for cell anchoring and interactions [35]. Interconnectivity is important because, after initial cell attachment, cells can migrate through the fibrillar spaces to access nutrients and metabolites necessary for their survival [35].

The mechanical properties of the PCL/PEO meshes depended on the polymer ratio. The modulus of elasticity of the fibrillar layer increased in the groups with 90 % and 50 % PEO (p < 0.042), with no difference among the polymer blends (p > 0.067). Notably, the 50/50 PCL/PEO formulation resulted in an elastic modulus (~20 MPa) that falls between the stiffness of dental pulp tissue (<10 kPa) and dentin (12–20 GPa) [36–40,73]. Tensile strength was significantly lower in the 90/10 group compared to the control (p = 0.037). Elongation at break was notably increased—about five times—in the 90/10 and 50/50 PEO groups, with no significant difference between them (p = 0.731; Fig. 2c). Surface wettability improved significantly in all PCL/PEO blends (p < 0.0001) compared to PCL fibers (control), with the highest hydrophilicity seen in the 50/50 formulation (Fig. 2d and e).

**Fig. 3.**
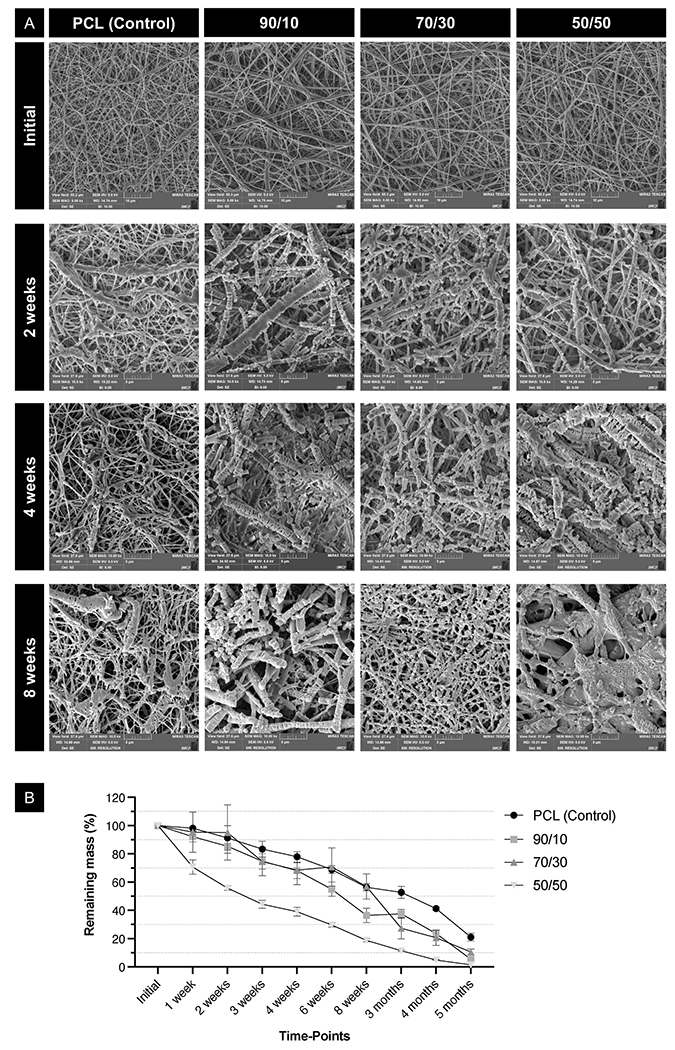
PEO modulates PCL fibers’ degradation profile. **(A)** Representative SEM images of the surface of the scaffolds before being placed in a lipase-containing (150 U/L) PBS solution and after 2, 4, and 8 weeks of enzymatic (lipase) degradation. **(B)** Remaining dry mass of the scaffolds up to 5 months of challenge using the lipase-containing enzymatic degradation solution. Mean and confidence intervals (95 % CI, n =6).

After 8 weeks of enzymatic challenge, the 50/50 PCL/PEO composition shows greater morphological disorganization compared to the control (PCL fibrillar mesh) or other polymeric blends (Fig. 3a). The enzymatic degradation assay enabled us to observe differences in degradation profiles by measuring changes in dry mass. Compared to the control (PCL fibrillar mesh), which retained the highest mass, all blends were more susceptible to degradation. Among these, the 50/50 composition was the most impacted by the enzymatic challenge (Fig. 3b). Enzymatic degradation of PCL by lipase involves hydrolysis primarily targeting the amorphous regions of the polymer, leading to surface erosion and increased crystallinity of the remaining material [41–43]. Using enzymatic degradation highlights differences in the degradation profiles of these polymeric blends in a shorter time frame. Understanding these mechanisms can help optimize PCL clinical applications, especially in controlled drug release and tissue engineering, aligning with the target tissue regeneration rate [41,44]. Although mechanical strength and degradation are often inversely related, PEO-containing formulations, particularly 50/50, exhibited both higher tensile strength and faster degradation. This may be due to improved fiber formation and plasticity provided by PEO, resulting in scaffolds that are structurally resilient yet more resorbable. Additionally, blending PEO with PCL can accelerate PCL degradation mainly because of increased hydrophilicity and structural changes that promote water uptake and hydrolysis [44].

**Fig. 4.**
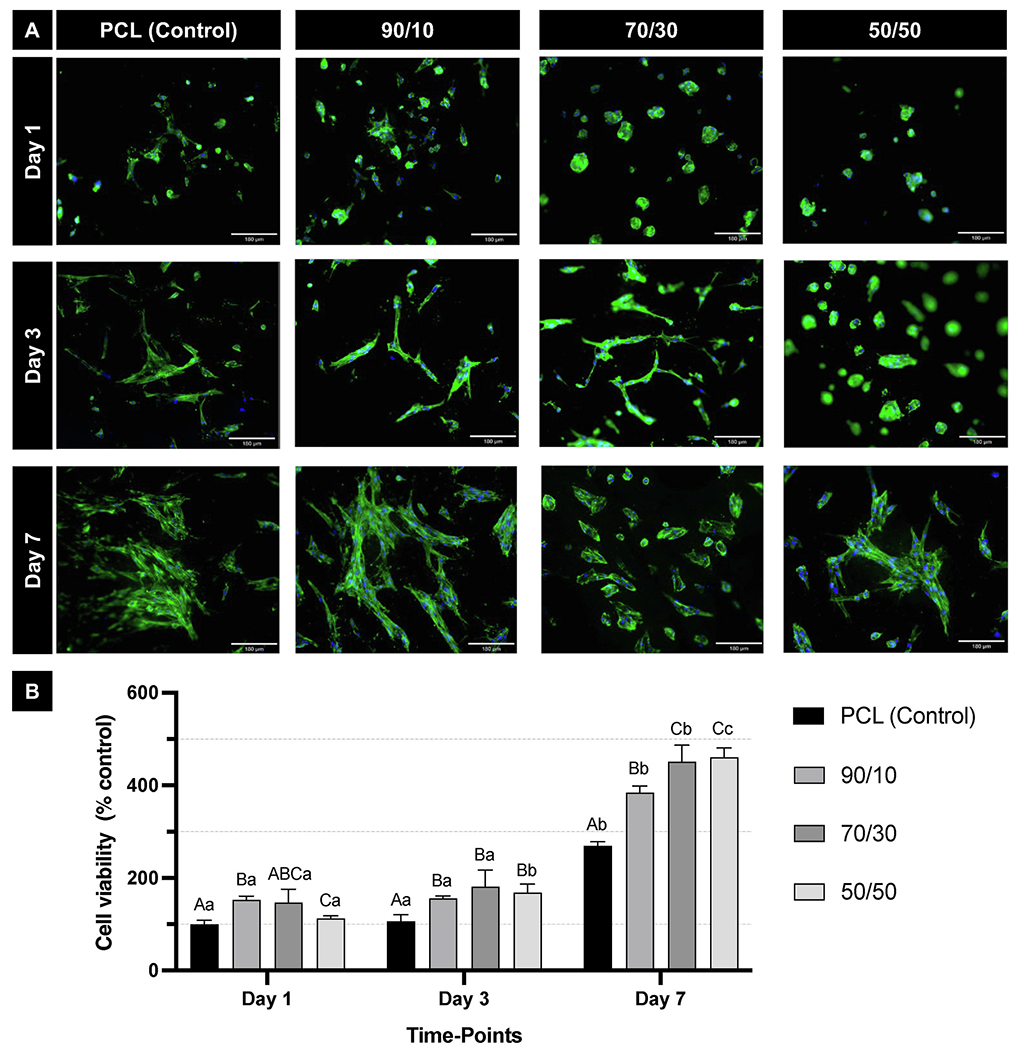
PCL/PEO fibers promote hDPSCs’ viability *in vitro*. **(A)** Adhesion and spreading of hDPSCs seeded on the surface of experimental scaffolds (10 ×). Actin filaments (green) were stained with ActinGreen 488 reagent, and cell nuclei (blue) were stained with DAPI. Scale bar: 180 μm. **(B)** Cell viability of hDPSCs seeded on the surface of experimental scaffolds. Mean and standard deviation. Values calculated considering the PCL group (control) at day 1 as 100 %. Capital letters compare the main effect “Groups” while lowercase letters compare the main effect “Time-points”. Different letters denote statistically significant differences (repeated measures ANOVA/Sidak; n = 6; α = 5 %). (For interpretation of the references to color in this figure legend, the reader is referred to the Web version of this article.)

All groups exhibited similar cell adhesion and spreading during the evaluated periods (Fig. 4a). Cell viability increased for all polymer blends compared to pure PCL fibers at 3 and 7 days (p < 0.035). After 7 days, the 70/30 and 50/50 compositions showed comparable cell viability (p = 0.995), likely due to the higher PEO content in both blends (Fig. 4b). As the increased proportion of PEO enhances the scaffold’s hydrophilicity, it may also improve nutrient diffusion, thereby promoting better cell adhesion and proliferation [45]. This could explain the similar and higher cell viability observed in these groups at day 7 compared to PCL. Additionally, all groups demonstrated a significant increase in cell viability over time (p < 0.0001), indicating good overall cytocompatibility of the fibrillar meshes [45]. The use of PCL to fabricate scaffolds for regenerating mineralized tissues has been extensively studied [20–22,46,47]. However, the use of this polymer limits its complete replacement by regenerated tissue due to its slow biodegradability [18]. Herein, various polymeric blends of PCL and PEO were used to create the fibrillar layer to tailor PCL’s degradation rate. Combining hydrophilic polymers can modulate the physical and mechanical properties of scaffolds, allowing for adjustment of each polymer’s amount to achieve properties suited to the tissue being regenerated [48]. The blend of PCL with 50 % by volume of PEO resulted in increased wettability, improved mechanical properties, and a faster degradation rate, which becomes more clinically relevant considering the time needed for the synthesis of a mineralized tissue barrier (~45–60 days) to seal a pulp exposure [49].

### 2.3. Incorporation of calcium hydroxide (CH) into the PCL/PEO fibrillar layer

**Fig. 5.**
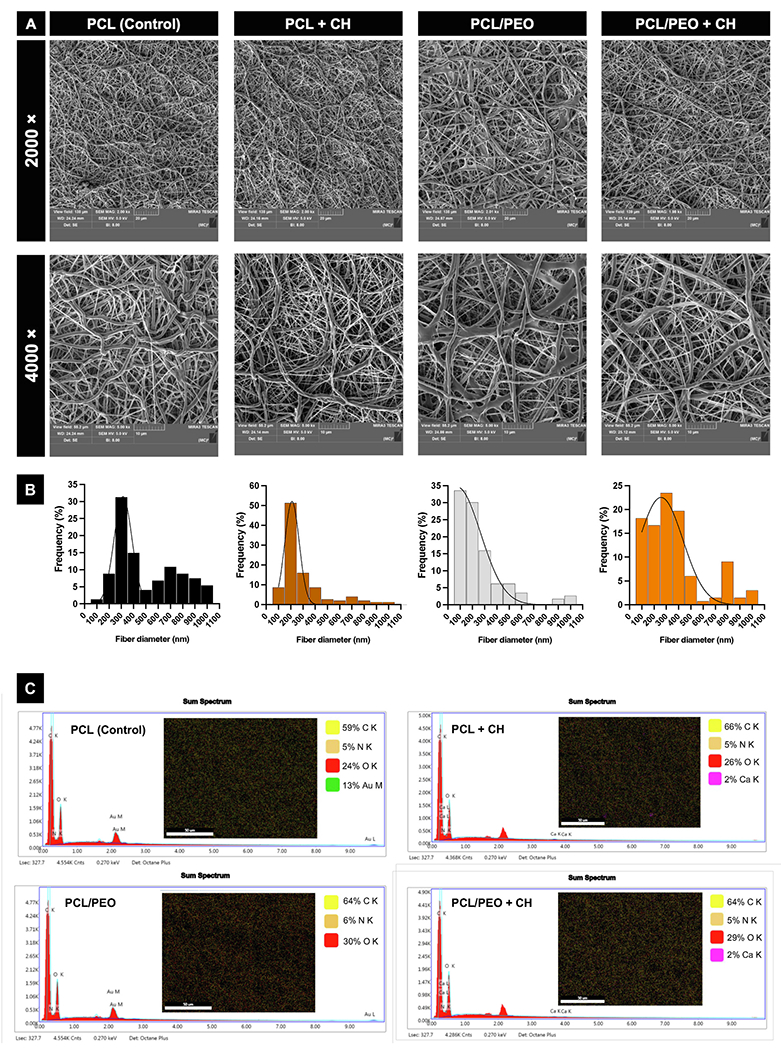
Morphological characterization of the PCL/PEO fibrillar layer incorporated with CH. **(A)** Representative SEM images of the surface of fibrillar scaffolds (2000 ×and 5000 ×). **(B)** Fiber diameter frequency plots of the experimental scaffolds (n =150). **(C)** Spectrograms generated by energy dispersive X-ray (EDS) show the presence or absence of peaks related to the element Ca from the calcium hydroxide incorporated in the PCL/PEO fibers. Inserts show EDS mapping. Peaks associated with Au represent the presence of Au due to sample metallization.

The 50/50 ratio was chosen for combination with CH due to its positive effects on wettability and cell viability. To assess the influence of CH incorporation in the 50/50 polymer blend, pure PCL fibers and CH-loaded PCL fibers were used as control groups (PCL, PCL + CH, PCL/PEO, and PCL/PEO + CH). Incorporating 0.2 % CH was selected because it preserved the morphological characteristics of the fibrillar layer. Higher CH concentrations disrupted consistent fiber formation (Supplementary Material). SEM images confirmed the formation of uniform, randomly distributed fibers in all electrospun fibrillar mesh formulations (Fig. 5a). Fiber diameter analysis showed a higher frequency of 300 nm fibers for pure PCL, which decreased to 200 nm with the addition of CH. In the fibrillar mesh made from the polymer blend, a broader range of fiber diameters was observed (between 100 and 300 nm), which extended to 100–400 nm with added CH (Fig. 5b). SEM images and fiber diameter measurements indicated that, despite changes in the fiber diameter distribution for meshes from the polymer blend, all formulations had a similar, uniform morphology across the mesh, regardless of CH addition. The presence of CH in the polymeric fibers (pure PCL and 50/50 PCL/PEO blend) was confirmed by energy dispersive spectroscopy (EDS). Elemental mapping showed CH distribution across the fiber surface, with a total calcium content of 2 % identified in the mapping, regardless of the fiber’s polymeric composition (Fig. 5c). Additionally, calcium ions were released, confirming their presence in the fibrillar layer. The calcium release data align with scaffolds that include inorganic calcium-containing particles at similar concentrations used here [20–22,50].

**Fig. 6.**
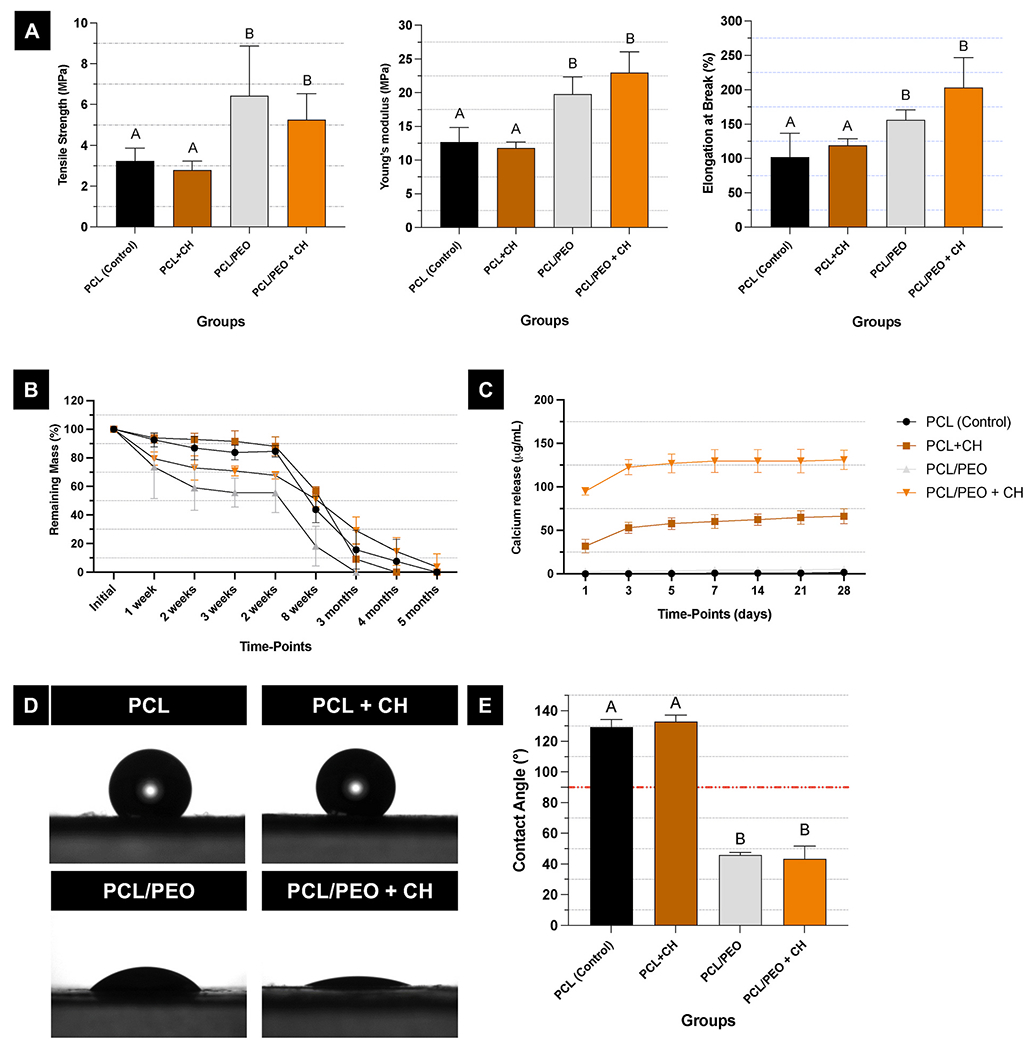
Mechanical and physical characterization of the PCL/PEO fibrillar layer incorporated with CH. **(A)** Graphs displaying mechanical properties of the experimental scaffolds. Mean and standard deviations. Different letters denote statistically significant differences (ANOVA and Welch’s ANOVA/Tukey or Games-Howell; n = 6; α = 5 %). **(B)** Remaining mass of the experimental scaffolds in lipase-containing (150 U/L) PBS. Mean and 95 % confidence intervals (n =6). **(C)** Calcium release from experimental samples in ultrapure water. Mean values and 95 % confidence intervals (n =4). **(D)** Images of a 1 μL water droplet on the surface of the scaffolds. **(E)** Bar graphs representing the contact angle between the ultrapure water droplet and the surface of the scaffolds. Mean and standard deviation. Different letters indicate statistically significant differences between groups (ANOVA/Tukey; n = 6; α = 5 %). The horizontal red dashed line indicates the 90°limiting contact angle between the hydrophobic and hydrophilic properties. (For interpretation of the references to color in this figure legend, the reader is referred to the Web version of this article.)

Tensile testing showed an increase in the elastic modulus for the fibrillar mesh made from the polymeric blend, both with and without CH addition, compared to the control group (p < 0.0001; Fig. 6a). The blend’s tensile strength was about twice that of pure PCL (p < 0.0001; Fig. 6a). A similar pattern was seen for elongation at break, with the polymeric blend exhibiting a higher percentage of elongation (p ≤ 0.041), regardless of CH addition (p ≥ 0.149; Fig. 6a). Adding CH did not impact the elastic modulus, tensile strength, or elongation at break, no matter the polymeric composition (p ≥ 0.149; Fig. 6a). When used clinically, such as in direct pulp capping and pulpotomies, these polymeric nanofibers will be covered with a lining or restorative material. Therefore, they need to have enough mechanical strength to support the restoration and meet the functional demands on the dental element in the oral cavity until replaced by mineralized tissue. In this study, the PCL/PEO polymeric blends led to scaffolds with significantly better mechanical properties compared to scaffolds made from pure PCL, especially for the 50/50 PCL/PEO blend. Of note, adding CH did not alter the modulus of elasticity (stiffness), maximum tensile strength, or elongation at break. It is also important to highlight that these improvements in mechanical properties did not affect the mass loss of the fibrillar layer; that is, the degradation rate of the PCL/PEO fibrillar layer remained higher than that of scaffolds made solely from pure PCL.

The enzymatic degradation assay showed that the PCL/PEO blend degraded faster than fibers made from pure PCL. The polymer blend achieved complete degradation in a lipase-rich solution (150 U/L) after 12 weeks, whereas pure PCL took 20 weeks. The presence of CH in the PCL/PEO fibrillar layer provided greater stability during degradation after the first 4 weeks (Fig. 6b). However, the scaffolds mainly released the incorporated calcium during the initial 7 days, then plateaued regardless of the polymer composition. In contrast, the fibrillar layer made from the polymer blend released 2.5 times more calcium overall than fibers made of pure PCL (Fig. 6c). The blend improved degradation and calcium release because of increased hydrophilicity. CH quickly dissociates into Ca^2+^ and OH^−^ in water [51], causing rapid calcium ion release in the first days, especially with the polymer blend fibers. Slow-degrading polymers like polyesters can trap some particles within the matrix [52], making them difficult to release and limiting cell response. In our system, partial dissociation of Ca(OH)_2_ likely occurs during electrospinning, and the OH^−^ ions could react further with the polymer, such as hydrolyzing ester bonds in PCL, which can reduce the burst release of hydroxyl ions. This process results in a more gradual and buffered ion release profile. Thus, the polymer matrix functions both as a physical barrier and a chemical modulator, reducing the risk of sudden pH spikes while providing sustained Ca^2+^ availability, key for eliciting the desired biological responses.

The contact angle exhibited by the PCL/PEO fibrillar layer was below the 90° threshold, demonstrating the blend’s effectiveness in altering the hydrophobic behavior of PCL (Fig. 6d and e). The addition of inorganic particles into PCL can modify the physical and mechanical properties of fibrous scaffolds; these alterations in the supporting structure also serve as signals for cell differentiation [16,53]. However, the CH concentration used in this study did not influence the mechanical properties or wettability. The fibrillar layer formed from the polymeric blend showed a significant decrease in the contact angle (p < 0.0001) between the water droplet and the fiber’s surface, with no effect from the CH incorporation (p ≥ 0.647).

**Fig. 7.**
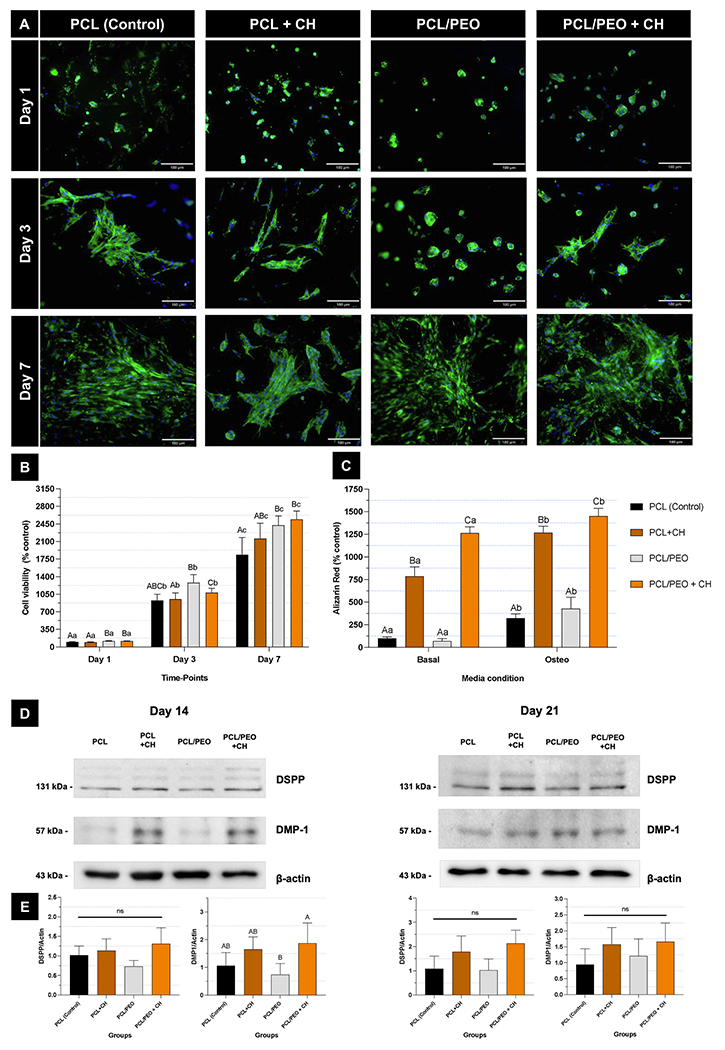
CH incorporation in PCL/PEO fibrillar layer promotes hDPSCs differentiation. **(A)** Adhesion and spreading of hDPSCs seeded on the surface of experimental scaffolds (10 ×). Actin filaments (green) were stained with ActinGreen 488 reagent, and cell nuclei (blue) were stained with DAPI. **(B)** Cell viability of hDPSCs seeded on the scaffolds’ surface. Mean and standard deviations. Values calculated considering the PCL group (control) at day 1 as 100 %. Capital letters compare the main effect “Groups” while lowercase letters compare the main effect “Time-points”. Different letters denote statistically significant differences (Repeated measures ANOVA/Sidak; n = 6; α = 5 %). **(C)** Mineralized matrix deposition by hDPSCs seeded on scaffolds. Mean and standard deviations. Values calculated considering the PCL group (control) at day 1 in basal medium as 100 %. Capital letters compare the main effect “Groups” while lowercase letters compare the main effect “Media condition” (two-way ANOVA/Sidak; n = 6; α = 5 %). **(D)** Western blot of DSPP and DMP-1 by hDPSCs seeded on the scaffold’s fibrillar surface after 14 and 21 days of culture. **(E)** Band intensities of Western blot analysis measured using ImageJ software. Different letters indicate statistically significant differences between groups. ns: non-significant differences (ANOVA/Tukey; n = 4; α = 5 %). (For interpretation of the references to color in this figure legend, the reader is referred to the Web version of this article.)

Fluorescence images of cells in contact with the fibrillar layer of the scaffolds showed uniform cell adhesion and increased cell spreading over time across all evaluated groups (Fig. 7a). This was confirmed by the cell viability assay (Fig. 7b). Notably, when examining the effect of the “time point” factor, all groups exhibited an increase in cell viability over time (p ≤ 0.008). Analysis of the “Group” variable revealed that fibers made from the polymer blend showed higher cell viability after 7 days of culture (p ≤ 0.037) compared to the pure PCL (control). The PCL + CH group displayed cell viability similar to that of the polymeric blend during the later evaluation period (p ≥ 0.216; Fig. 7b). Incorporating CH into the fibers positively impacted mineralized matrix production, regardless of the fiber’s polymeric composition and the use of differentiation culture medium (p ≤ 0.001; Fig. 7c). For the polymer blend, adding CH enhanced DMP1 synthesis after 14 days *in vitro* (p = 0.044). However, DSPP and DMP1 synthesis showed no statistical differences among groups after 21 days (p ≥ 0.058; Fig. 7d and e). These results suggest that the CH concentration chosen supported an early odontogenic response without compromising cell viability. Although we did not explore different CH concentrations, literature indicates that low extracellular calcium levels can stimulate human dental pulp cells proliferation [54] and activate calcium-mediated signaling pathways involved in mineralized tissue formation [50,55–57]. Conversely, high levels of Ca^2+^ and OH^-−^ release may raise local pH, causing toxicity and tissue necrosis [58]. Our findings show that the calcium release profile achieved with 0.2 % CH was adequate to promote early DMP1 expression while preserving cytocompatibility.

### 2.4. In vivo evaluation of the bilayer polymeric scaffold

**Fig. 8.**
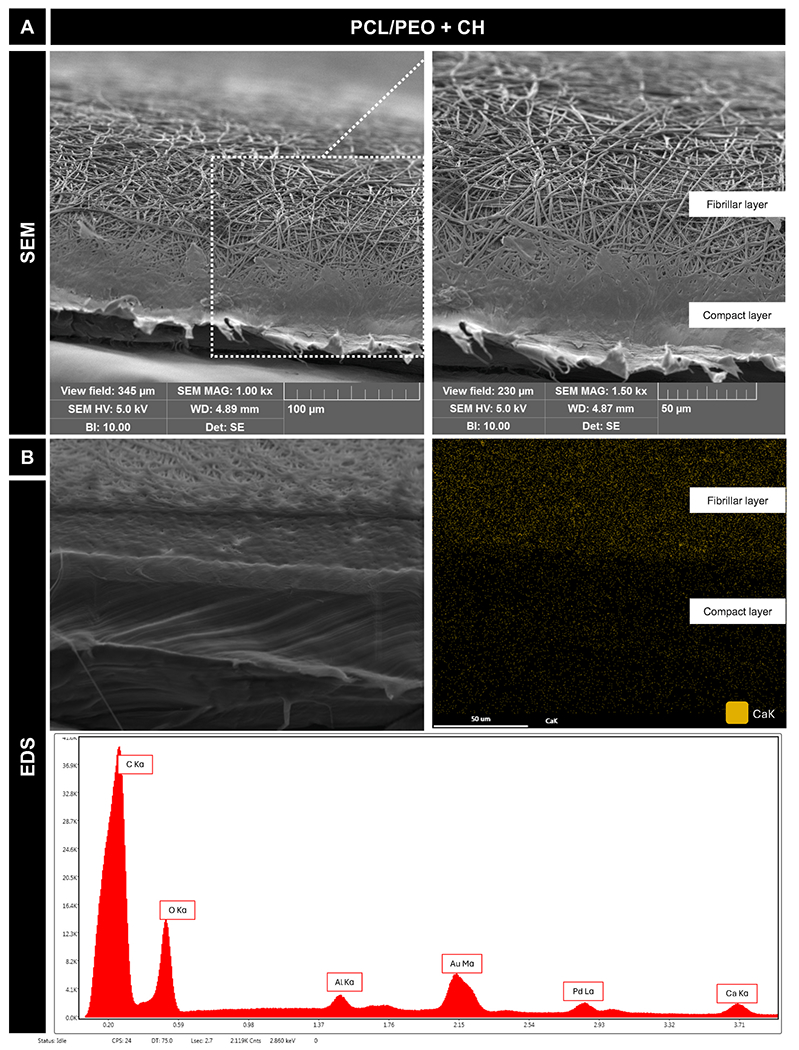
Morphological characterization of the bilayer scaffold incorporated with CH. **(A)** Exemplary SEM images of the cross-section of the scaffolds (1000 ×and 1500 × ). **(B)** EDS mapping for calcium and spectrogram generated by energy dispersive X-ray (EDS) shows the presence of calcium (Ca) from the CH incorporated in the fibrillar layer. Peaks associated with Au and Pd are indicative of sample metallization before imaging. Al presence denotes the composition of the sample stub.

The cross-sectional characterization of the bilayer scaffold composed of a compact layer and a fibrillar electrospun layer containing calcium hydroxide (CH) was assessed using SEM. SEM images (Fig. 8a) clearly show the interface between the dense compact layer and the porous fibrillar layer, demonstrating the integrity and structural organization of the bilayer design. Elemental mapping via EDS (Fig. 8b) reveals a sitespecific distribution of calcium (Ca) exclusively within the fibrillar layer, confirming the successful incorporation of CH. The corresponding EDS spectrum (Fig. 8b) shows peaks for carbon and oxygen, representative of the PCL/PEO matrix, along with gold (Au) and palladium (Pd) from the sample’s conductive coating, and aluminum (Al) from the sample stub. The distinct Ca peak further validates the presence of calcium hydroxide within the scaffold. Together, these results confirm the bilayer design and targeted localization of CH within the fibrillar layer.

**Fig. 9.**
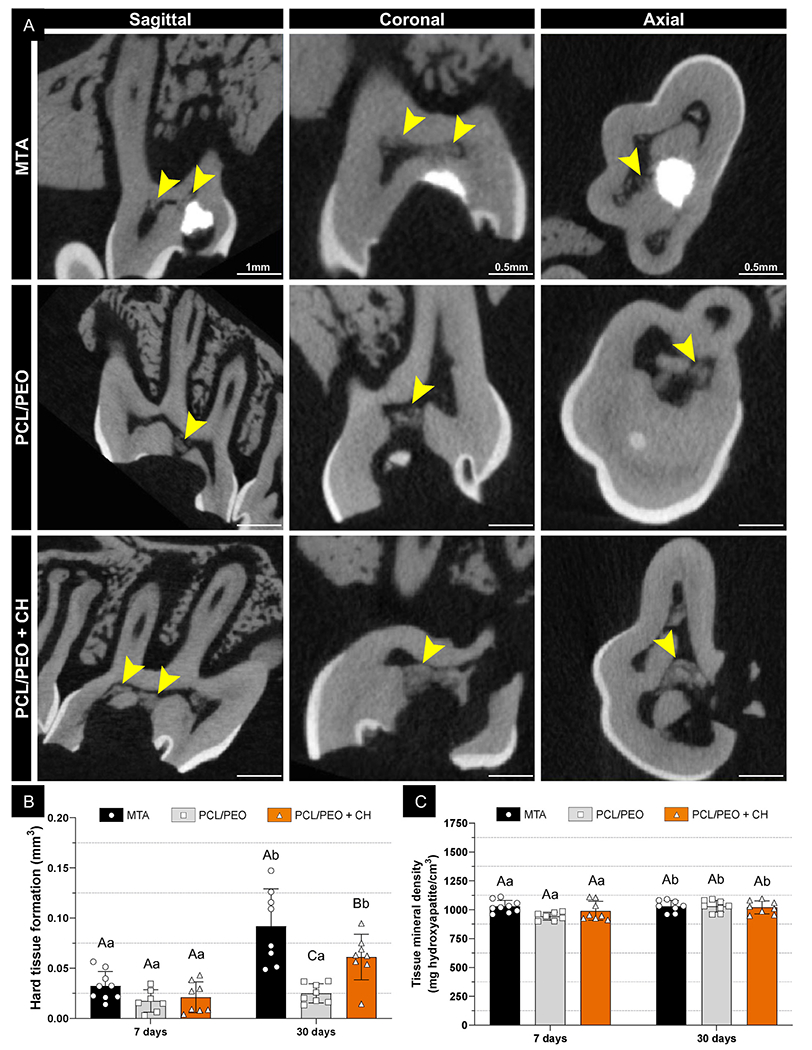
CH improves mineralized tissue formation for PCL/PEO bilayer scaffolds in a rat pulp capping model. **(A)** Micro-computed tomography (microCT) images after 30 days. The yellow arrowhead marks newly formed mineralized tissue in the evaluated region. Scale bars: 1 mm (sagittal view) and 0.5 mm (coronal and axial views). **(B)** Mineralized tissue formation (mm^3^) induced by each material (mean ±SD). **(C)** Tissue mineral density (mg hydroxyapatite per cm^3^) in the newly formed tissue (mean ±SD). Capital letters compare the main effect “Groups” while lowercase letters compare the main effect “Time-points”. Different letters denote statistically significant differences (Unbalanced two-way ANOVA/Sidak; n ≥ 7; α = 5 %). (For interpretation of the references to color in this figure legend, the reader is referred to the Web version of this article.)

For all *in vivo* analyses, MTA was used as a clinical control, while PCL/PEO (50:50) bilayer scaffolds with or without CH incorporation served as experimental groups to determine the effect of CH in the bilayer design. Micro-CT analysis of *in vivo* mineralized tissue formation showed a time-dependent increase in mineral deposition in the MTA and PCL/PEO + CH groups (Fig. 9). At seven days, the clinical control (MTA) exhibited slightly more mineralized tissue formation (0.032 mm^3^) compared to PCL/PEO (0.017 mm^3^) and PCL/PEO + CH (0.021 mm^3^); however, these differences were not statistically significant (p ≥ 0.1; Fig. 9b). The similar levels of mineral deposition at this early time-point were expected, as seven days is typically not enough to detect substantial mineralized tissue formation. By 30 days, all groups showed increased mineral deposition. MTA demonstrated nearly a threefold increase from its 7-day value (0.092 mm^3^, p < 0.0001), while PCL/PEO remained statistically similar to its 7-day measurement (0.025 mm^3^). In contrast, PCL/PEO + CH significantly increased, nearly tripling its initial value (0.061 mm^3^). Despite these improvements, MTA consistently outperformed the experimental groups, with mineralized tissue formation at 30 days being 3.7 times greater than PCL/PEO (p < 0.0001) and 1.5 times higher than PCL/PEO + CH (p = 0.015; Fig. 9b). Regarding the quality and density of the newly formed mineralized tissue, no significant differences were observed between the groups at the same time-points. This finding indicates that the mineralization induced by PCL/PEO + CH was of comparable quality to that promoted by MTA. However, while MTA demonstrated superior mineralization potential in terms of volume at both time-points, it is important to highlight the promising performance of PCL/PEO + CH (Fig. 9c). This material offers improved handling properties, which could help overcome some of MTA’s practical challenges, including its long setting time, difficult handling, and high cost [59].

**Fig. 10.**
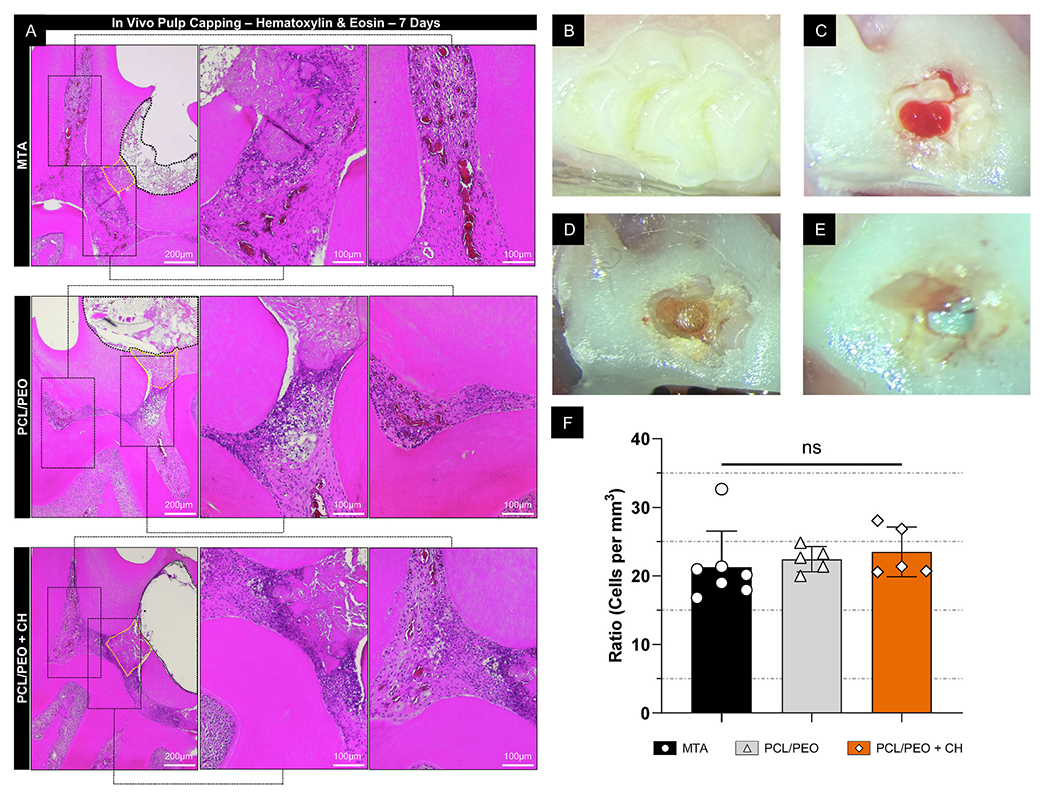
PCL/PEO + CH bilayer scaffolds present an inflammatory response similar to MTA in a rat pulp capping model. Seven days after the pulp capping procedure, panel **(A)** displays hematoxylin and eosin-stained images at a lower magnification (4 ×, scale bar: 200 μm) with black dotted rectangles indicating areas that were subsequently examined at higher magnification (10 ×, scale bar: 100 μm). The yellow dashed outline denotes the implanted material in contact with the pulp tissue, and the black dotted contour above indicates the space occupied by the glass ionomer. **(B)** The initial appearance of the upper first molar, while **(C)** shows the tooth after placement of the gingival barrier and pulp exposure. **(D)** The immediate view following the MTA application, while **(E)** shows the immediate view after placing the PCL/PEO + CH scaffold. **(F)** Indicates the ratio of cells (cells per total pulp area in mm^3^) for the three applied materials, with data presented as mean ± standard deviation (Unbalanced ANOVA/Tukey; n ≥ 5; α = 5 %) with “ns” indicating non-significant differences. (For interpretation of the references to color in this figure legend, the reader is referred to the Web version of this article.)

Histological analysis of H&E-stained sections at 7 days showed similar cellular responses across all groups, with the total cell density in the pulp tissue not significantly different between MTA (21.3 ± 5.3 cells/mm ^2^), PCL/PEO (22.4 ± 1.8 cells/mm ^2^), and PCL/PEO + CH (23. 5 ± 3.6 cells/mm ^2^; Fig. 10). Since rat molar teeth, including their pulp tissue, share anatomical, histological, biological, and physiological similarities with human molars [60], the lack of excessive cellular infiltration or differences in cell density suggests that PCL/PEO + CH induces an inflammatory response comparable to that of MTA, indicating it does not impair early pulp healing. An initial inflammatory response is vital for tissue repair and regeneration, during which pulp cells proliferate, increase in size and number, and undergo phenotypic changes to produce collagen and other molecules involved in mineralization [61]. Additionally, when pulp capping is performed, as necrotic tissue is metabolized, granulation tissue formation — characterized by proliferating capillaries and fibroblasts — marks the early stages of pulp tissue repair. Because this cellular reaction is critical for wound healing, our results suggest that PCL/PEO + CH supports this biological process similarly to MTA. Furthermore, incorporating CH into the fibrillar layer of our engineered scaffold enhanced the biological response, as demonstrated by increased cell viability and morphology compatible with odontoblast-like cells. This corroborates previous studies showing that low concentrations of CH (1–10 μg/mL) assist odontoblastic differentiation [62], supporting clinical evidence of hard tissue bridge formation [63]. Since inflammation in the pulp is often caused by cytotoxic effects of the capping material or bacterial infiltration from microleakage, the similar inflammatory response seen across all groups further confirms the biocompatibility of PCL/PEO + CH. Along with the long-term micro-CT results, these histological findings suggest that the mineralization potential of the bilayer scaffold is clinically promising, strengthening its potential as an alternative to MTA for vital pulp tissue therapy.

**Fig. 11.**
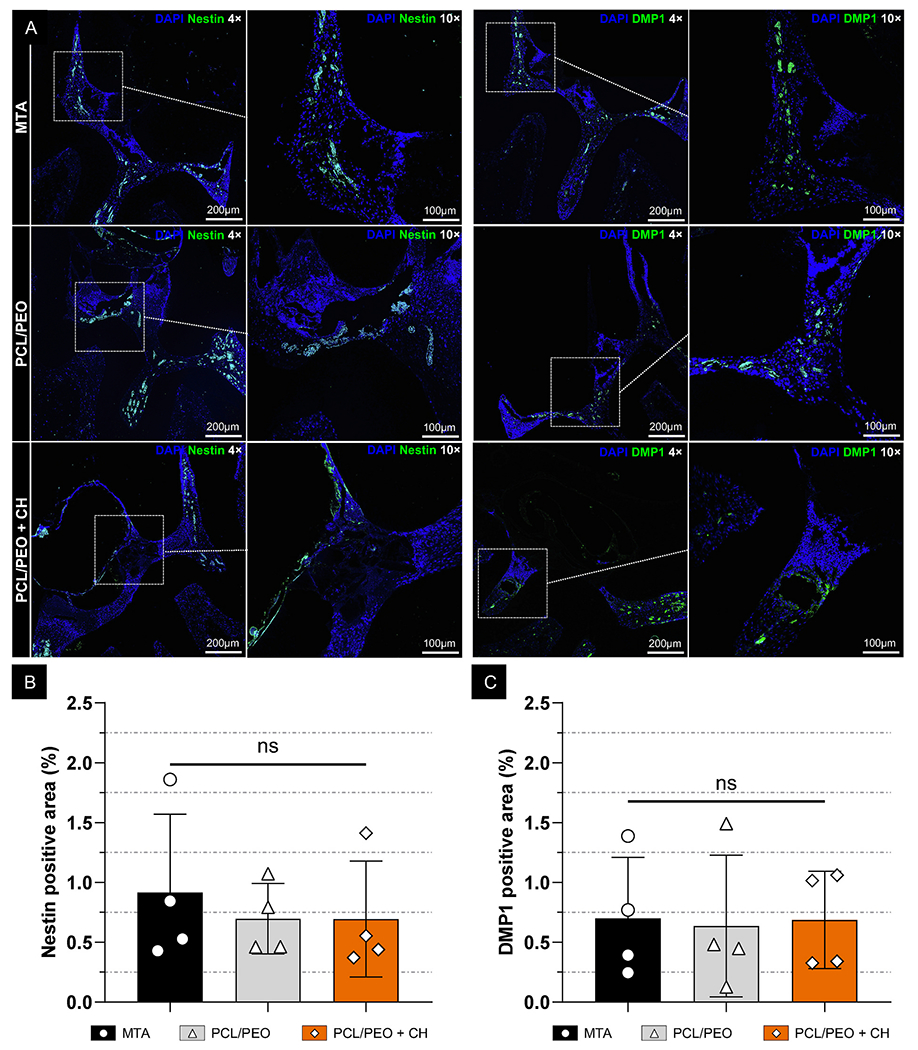
PCL/PEO +CH bilayer scaffolds present dentinogenic response similar to MTA in a rat pulp capping model. *In vivo* immunolabeling of dentinogenic markers Nestin and DMP1 was performed using Alexa Fluor 488 (green), 7 days after pulp capping. **(A)** Images were captured at 4 × (scale bar: 200 μm) and 10 × (scale bar: 100 μm) magnifications, with the white dotted square indicating the region selected for higher magnification. **(B–C)** Graphs show the percentage of Nestin and DMP1 immunolabeled positive area quantified using ImageJ. Data are presented as mean ± SD, with “ns” indicating non-significant differences. (ANOVA/Tukey; n = 4; α = 5 %). (For interpretation of the references to color in this figure legend, the reader is referred to the Web version of this article.)

Nestin and DMP1 are critical markers in pulp repair and dentinogenesis due to their connection to odontoblast differentiation and mineralized tissue deposition. Nestin is a well-established marker of odontoblast-like cell activity, reflecting their differentiation and functional maturation during reparative dentin deposition [64,65]. Conversely, DMP1 (Dentin Matrix Protein 1) plays a vital role in dentin mineralization by regulating hydroxyapatite crystallization and promoting the formation of mineralized tissue [66,67]. In our study, immunofluorescence analysis (Fig. 11a) showed no significant differences in the expression levels of these markers among the three experimental groups. Nestin expression was found to be 0.92 % ± 0.65 % in the MTA group. In comparison, PCL/PEO and PCL/PEO + CH exhibited slightly lower values at 0.70 % ± 0.29 % and 0.69 % ± 0.48 %, respectively (Fig. 11b). Similarly, DMP1 expression remained comparable across the groups, with MTA at 0.70 % ± 0.51 %, PCL/PEO at 0.64 % ± 0.59 %, and PCL/PEO + CH at 0.69 % ± 0.41 %. These results suggest that the PCL/PEO bilayer scaffold loaded with calcium hydroxide supports odontoblast-like activity and mineralization similarly to MTA (Fig. 11c).

**Fig. 12.**
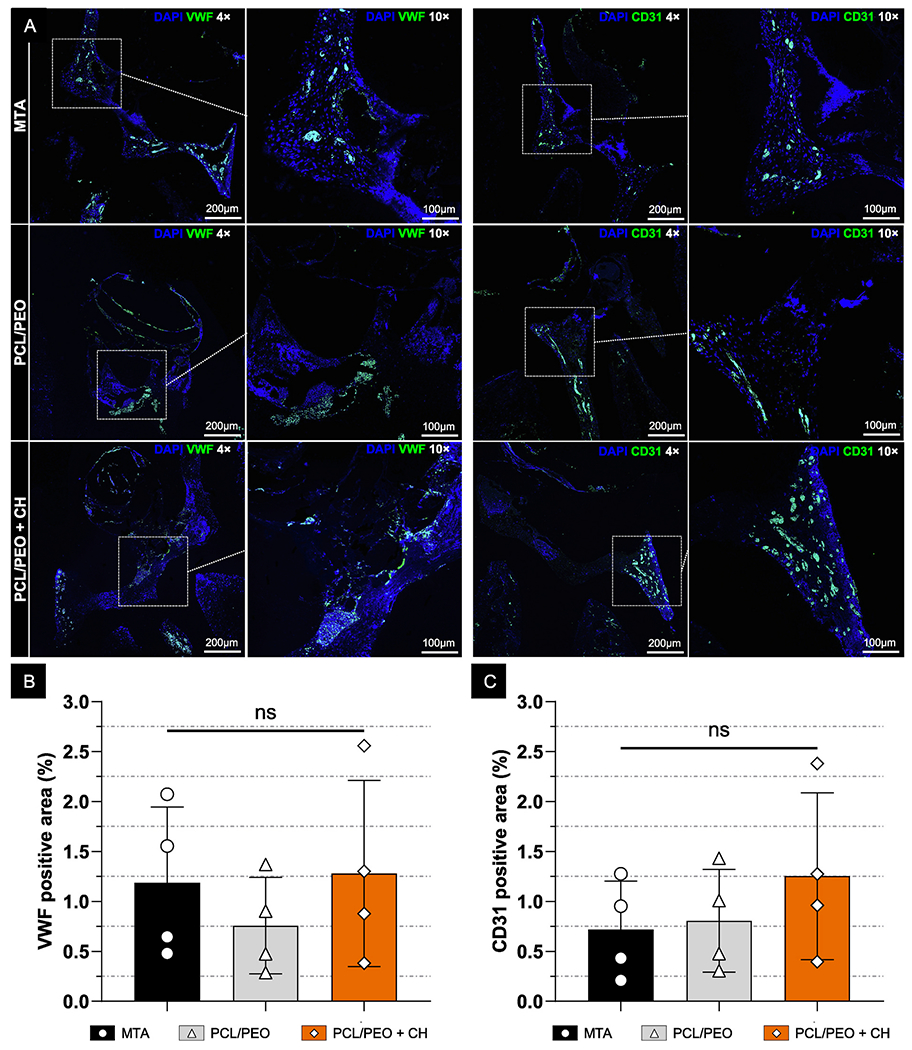
PCL/PEO + CH bilayer scaffolds present an angiogenesis response similar to MTA in a rat pulp capping model. *In vivo* immunolabeling of angiogenesis markers vWF and CD31 was performed using Alexa Fluor 488 (green), 7 days after pulp capping. **(A)** Images were captured at 4 × (scale bar: 200 μm) and 10 × (scale bar: 100 μm) magnifications, with the white dotted square indicating the region selected for higher magnification. **(B–C)** Graphs show the percentage of vWF and CD31 immunolabeled positive area quantified using ImageJ. Data are presented as mean ± SD, with “ns” indicating non-significant differences. (ANOVA/Tukey; n = 4; α = 5 %). (For interpretation of the references to color in this figure legend, the reader is referred to the Web version of this article.)

Cluster of Differentiation 31 (CD31) is a transmembrane protein that regulates cellular adhesion, leukocyte migration, platelet and T-cell activation, and angiogenesis. In pulp tissue, it is primarily expressed by endothelial cells, playing a key role in inflammation through cell diapedesis. Endothelial cells also regulate coagulation, thrombolysis, and vascular permeability to facilitate effective wound healing. Similarly, von Willebrand factor (vWF), a glycoprotein secreted by these cells, is crucial for hemostasis and platelet adhesion at sites of vascular injury. Together, CD31 and vWF serve as markers of endothelial function and vascularization, both of which are essential for tissue repair and regeneration [68–71]. In this study, CD31 expression was slightly higher in PCL/PEO (0.81 ± 0.51 %) compared to MTA (0.72 ± 0.49 %), with PCL/PEO + CH showing the highest value (1.25 ± 0.83 %; Fig. 12c). Likewise, vWF expression was strongest in the PCL/PEO + CH group (1.28 ± 0.93 %), followed by MTA (1.19 ± 0.76 %) and PCL/PEO (0.76 ± 0.48 %; Fig. 12b). Although these expression levels showed an upward trend for the PCL/PEO + CH bilayer scaffolds, no significant differences were found among the groups (Fig. 12a). Thus, our biomaterial performed similarly to the clinical standard (MTA) in promoting endothelial marker expression.

The findings gathered from this investigation were conducted under controlled laboratory conditions that may not fully mimic the complex biological environment of human dental tissues, which could influence how our results might translate to clinical settings. While *in vitro* experiments offer valuable insights into material properties, degradation rates, and cytocompatibility, they do not account for the dynamic interactions with immune responses, vascularization, and long-term functional integration *in vivo*. Additionally, the rat molar model, although widely used due to its anatomical and biological similarities to human teeth, has inherent species differences. The healing process in rat molars is histologically comparable to that in humans, but technical challenges, such as the small size of rat teeth, must be considered. This study also focused on short-to mid-term evaluations, leaving open questions regarding the long-term stability, degradation kinetics, and potential adverse effects of polymeric blends in a clinical setting. Furthermore, the impact of mechanical loading and bacterial contamination, which are critical factors in a real oral environment, was not fully assessed in this study. These limitations highlight the need for further long-term, clinically relevant investigations to validate the safety and efficacy of the developed PCL/PEO + CH bilayer scaffolds as a viable alternative for vital pulp therapy.

In summary, the 50:50 PCL/PEO bilayer scaffold with 0.2 % CH promoted mineralized matrix formation and maintained pulp cell viability despite the cytotoxic effects of traditional glass ionomer cement used as a liner material. Although mineralization was less than that of MTA, the bilayer scaffold elicited similar inflammatory and molecular responses involved in dentinogenesis and angiogenesis. These findings support the *in vitro* results, where improved hydrophilicity and calcium release from the bilayer scaffold enhanced hDPSC viability and differentiation. With easier handling and lower cost, this bilayer scaffold could serve as a practical alternative to MTA.

## 3. Methods

### 3.1. Synthesis and characterization of the compact layer

Polycaprolactone solutions (PCL; 10 % w/v; Sigma-Aldrich, St. Louis, MO, USA) were prepared in chloroform (Merck, Darmstadt, Germany) and kept under magnetic stirring at room temperature (RT) for 30 min, in 10 mL glass beakers with a base area of 6.15 cm^2^. The beakers containing different volumes of this solution (600, 800, and 1000 μL) were placed in a vacuum oven for 3 h to eliminate bubbles formed during solvent evaporation. Afterwards, the beakers were kept at RT for 24 h to ensure complete evaporation of the solvent.

#### 3.1.1. Morphological characterization of the compact layer

To evaluate the compact layer, 3 mm specimens were obtained using a dermatological biopsy punch (Acu-Punch, Fort Lauderdale, FL, USA). For thickness evaluation, the compact layers were submerged in liquid N_2_ for 30 s and then fractured to improve visualization of the cross-section. The specimens were mounted on metal stubs, coated with gold, and examined using a scanning electron microscope (SEM; JMS-6610V, JEOL, Tokyo, Japan). Surface analysis (at 1000× and 3000 × magnification) and cross-sectional analysis (at 300 × magnification) were performed to assess the thickness uniformity at four randomly selected regions of each sample (ImageJ software; NIH, National Institute of Health, Bethesda, MD, USA). Additionally, the surface homogeneity and presence of porosities were studied.

#### 3.1.2. Cytocompatibility on hDPSCs and sealing potential in an artificial pulp chamber model

For the following analyses, a culture of human dental pulp stem cells (hDPSCs; Lonza Inc; #PT-5025; Walkersville, MD, USA) grown in α-MEM culture medium supplemented with 15 % fetal bovine serum (FBS) and 1 % penicillin/streptomycin (all from Gibco) at 37 °C with 5 % CO_2_ between the 3rd and 8th passage was used. After forming the compact layer with different thicknesses (PCL 600, PCL 800, and PCL 1000), specimens with an 8 mm diameter were prepared. These specimens were adapted to artificial pulp chambers (APCs) and secured with silicone rings (Fig. 1c), then sealed peripherally with a light-cured gingival barrier (Top dam, GM, Dentscare LTDA, SC, Brazil). Each compact layer/APC assembly was sterilized with UV light for 40 min, placed in a compartment of a 24-well plate, and 1 mL of α-MEM was slowly added, taking care to avoid air bubbles (Fig. 1c). One side of the compact layer contacted the culture medium, while the opposite side was exposed to the cavity lining material. Applying the cavity lining materials on the upper surface mimics clinical use over pulp capping agents, allowing the assessment of the scaffold’s sealing ability and its effectiveness in protecting the underlying pulp tissue from potentially cytotoxic components released by these liners.

For the groups related to the lining material, two different liners were used: a conventional glass ionomer cement (Ketac Molar Easymix, 3 M ESPE, Seefeld, Germany) and a resin-modified glass ionomer cement (Vitrebond, 3 M ESPE, St. Paul, MN, USA). The cements were prepared according to the manufacturer’s instructions. The freshly prepared cements (150 mg) were applied on top of the compact layer. For the resinmodified glass ionomer cement, photoactivation was also performed for 20 s with an LED light device (Valo, Ultradent, South Jordan, UT, USA), and the irradiance was measured immediately before use. After 24 h to allow diffusion of the lining material components, the culture medium was collected (extract), and 100 μL was applied to hDPSC previously seeded in 96-well plates (1 × 10^4^ cells/well) and maintained for 24 h at 37 °C with 5 % CO_2_ for initial adhesion. After 24 h of contact with the cells, the extracts were aspirated and discarded, and the cells were kept in culture medium with 10 % fetal bovine serum (FBS) until the designated time points for cell viability analysis.

At 1, 3, and 7 days after contact with the extracts, the culture medium was aspirated and discarded. The cells were then incubated in α-MEM without FBS, supplemented with alamarBlue (10 %; Invitrogen), for 3 h at 37 °C and 5 % CO_2._ The fluorescence of the solution was measured (560 nm excitation and 590 nm emission; Synergy H1; Hybrid Multi-mode Microplate Reader, BioTek; Winooski, VT, USA). The fluorescence values were converted into percentages, using the mean fluorescence of the control group (PCL 600) on day 1 as 100 %. Based on these results, the thinnest compact layer with the thinnest was selected for association with the fibrillar layer of the bilayer scaffold.

### 3.2. Screening of PCL/PEO ratio to define the fibrillar layer polymer composition

#### 3.2.1 Preparation of PCL/PEO fibrillar layer

Polymeric solutions of PCL (10 %; w/v) in 1,1,1,3,3,3-Hexafluoro-2-propanol (HFIP) and poly(ethylene oxide) (PEO; 2 %; w/v; Sigma-Aldrich) in acetic acid were prepared separately and kept under magnetic stirring for 12 h. After complete dissolution of the polymers, different PCL/PEO (v/v) ratios were mixed (100/0; 90/10; 70/30; and 50/50) and stirred for an additional 3 h. The fibrillar layers were obtained via electrospinning. Each solution was transferred to a 3 mL syringe fitted with a 25G stainless-steel needle (CML Supply LLC, Lexington, KY, USA), connected to an automatic injection pump (KDScientific, Holliston, MA, USA) set to a flow rate of 0.4 mL/h. The high-voltage source (ES50P-10 W/DAM, Gamma High-Voltage Research, Inc., Ormond Beach, FL, USA) was adjusted to 20 kV, with a 25 cm distance between the needle tip and the collector. The collector, covered with aluminum foil, was rotated at 120 rpm. The resulting compact layers (10 × 10 mm) were placed in 24-well plates and sterilized using UV light for 1 h on each side, followed by an ethanol rinse (10 min) and two PBS washes. Additionally, the scaffolds were immersed in α-MEM for 1 h before being allocated to cell culture experiments.

#### 3.2.2. Morphological characterization of the fibrillar layer

The distinct electrospun fibrillar layer specimens (10 × 10 mm) were attached to metal stubs, coated with gold, and examined using a fieldemission scanning electron microscope (FEG/SEM, Tescan MIRA3 FEG-SEM, Tescan USA Inc., Warrendale, PA, USA). Images of the fibrillar surface of the samples were acquired at 2000 × and 5000 × magnifications with 12–15 kV. The fiber diameter was measured from three different areas of each sample at 5000 × magnification, with 50 nanofibers randomly chosen in each image to determine the diameter using ImageJ software.

#### 3.2.3. Mechanical properties

Uniaxial tensile tests were conducted on the scaffolds to determine their mechanical properties, using an eXpert 5601 mechanical testing machine (ADMET Inc., Norwood, MA, USA). Samples were rectangular (25 mm × 3 mm) and tested at a 1 mm/min rate with a 1 kN load cell (SM-250-961-250 lbf). The properties evaluated included tensile strength, modulus of elasticity, and elongation at break.

#### 3.2.4. Contact angle

The wettability of the scaffold’s fibrillar surface was analyzed by measuring the contact angle between the surface and deionized water using a goniometer (SCA20, DataPhysics Instruments GmbH, Filderstadt, BW, Germany). The samples (10 × 10 mm) were fixed in glass slides with a double-sided tape, making sure that there were no wrinkles or folded areas. A 1 μL droplet of deionized water was placed on the dry surface of each fibrillar layer. After 2 s, an image of the droplet was captured, and the angle between the droplet and the fibrillar surface was measured using the goniometer’s software (DROPimage Standard, Netcong, NJ, USA).

#### 3.2.5. Enzymatic degradation

The fibrillar layer (10 × 10 mm) had its dry mass measured on a high-precision balance (Microbalance XS106, Mettler-Toledo International Inc., Columbus, OH, USA) to determine the initial mass (M1). After the initial measurement, the samples were stored in glass tubes containing 5 mL of PBS supplemented with 150 U/L of Amano Lipase PS from *Burkholderia cepacia* (Sigma-Aldrich) and 0.02 % sodium azide (Sigma-Aldrich). The tubes were kept at 37 °C with agitation, and the solution was replenished every 2 days to maintain enzymatic activity. At each time-point, the samples were rinsed twice with deionized water and dried in a vacuum oven for 24 h. The samples were then weighed on a high-precision balance (M2) and subsequently immersed in PBS with lipase until the subsequent measurement. The fiber mass was recorded at 1, 2, 3, 4, 6, and 8 weeks, then monthly up to 5 months. Additionally, one sample was selected after 2, 4, and 8 weeks for SEM analysis to assess fibrillar morphology. The remaining mass at each time point was calculated using the following equation:

Eq 1 %remaining mass=M2/M1×100

#### 3.2.6. Cytocompatibility of PCL/PEO fibrillar layer

To assess cytocompatibility, 3 × 10^4^ dental pulp stem cells (hDPSCs) were seeded onto the fibrillar layer. The cell-seeded constructs were analyzed after 1, 3, and 7 days of incubation. At each time-point, the samples were washed with phosphate-buffered saline (PBS) and fixed in 4 % paraformaldehyde for 15 min at RT. After fixation, the cells were stained with ActinGreen^™^ 488 ReadyProbes^™^ Reagent (Invitrogen), a green fluorescent probe specific for actin filaments, diluted 1:20 in PBS, to visualize the cytoskeleton and evaluate cell spreading. Nuclear counterstaining was performed using DAPI (1:5000 dilution in PBS; Invitrogen). Fluorescent images were captured with a fluorescence microscope. At the same time-points, the culture medium was aspirated and discarded, and the cells were incubated with α-MEM without FBS, supplemented with alamarBlue (10 %; Invitrogen) for 3 h at 37 °C and 5 % CO_2_. Next, the supernatant was transferred to 96-well plates and analyzed using a spectrophotometer (560 nm excitation and 590 nm emission; Spectra Max iD3). The fluorescence values were converted into percentages, with the fluorescence of the control group (PCL) at day 1 set as 100 %.

### 3.3. Incorporation of calcium hydroxide (CH) into PCL/PEO fibrillar layer

#### 3.3.1. Preparation of PCL/PEO + CH fibrillar layer via electrospinning

After 12 h under magnetic stirring to ensure complete dissolution of the polymers, 0.2 % CH (w/v; Sigma-Aldrich) was added to the polymer blend and stirred for an additional 3 h. The fibrillar layers were electrospun following the same parameters mentioned in 3.2.1. Pure PCL fibers with or without CH incorporation were used as controls for all the following experiments.

#### 3.3.2. Morphological and chemical characterization of PCL/PEO + CH fibrillar layer

The scaffolds were cut (10 × 10 mm), fixed to metal stubs, coated with gold, and analyzed in a scanning electron microscope (SEM) as described in item 3.2.2. Additionally, the samples were analyzed in an EDS system (energy dispersive X-ray spectroscopy) to evaluate the CH incorporation.

#### 3.3.3. Mechanical properties, contact angle, and enzymatic degradation

These properties were tested following the protocols previously described in 3.2.3, 3.2.4, and 3.2.5.

#### 3.3.4. Calcium release

The scaffolds were immersed in 1 mL of ultrapure water (pH 7.0), and calcium release was measured at 1, 3, 5, 7, 14, 21, and 28 days. At each collection point, the ultrapure water in contact with the scaffolds (extract) was retrieved and transferred individually to 1.5 mL tubes, then stored at −20 °C. The stored extracts were thawed at room temperature and analyzed using a colorimetric calcium quantification kit (Sigma-Aldrich), following the manufacturer’s instructions. Calcium concentration was calculated using a calibration curve, and results were expressed in μg/mL.

#### 3.3.5. Cytocompatibility of PCL/PEO + CH fibrillar layer

The cytocompatibility of the scaffolds containing CH was tested following the protocol described in 3.2.6.

#### 3.3.6. Mineralized matrix formation

hDPSCs were cultured on the scaffolds in basal and differentiation (0.1 μM dexamethasone, 10 mM glycerophosphate, and 50 μg/mL ascorbic acid) α-MEM media for 21 days. They were fixed with 70 % ethanol at 4 °C and stained with Alizarin Red solution (40 mM, pH 4.2; Sigma-Aldrich). Then, cetylpyridine chloride solution (10 mM, pH 7.0; Sigma-Aldrich) was applied to dissolve the nodules, and the absorbance of the resulting solution was evaluated by spectrometry at 560 nm (Spectra Max iD3). The absorbance values were transformed into percentages, considering the absorbance of the control group (PCL) as 100%. The normalization of absorbance values was based on cell-free scaffolds maintained in culture for the same experimental period.

#### 3.3.7. Quantification of proteins related to odontogenic differentiation

To confirm the synthesis of proteins involved in odontogenic differentiation, cells seeded on the scaffold surfaces were lysed and stored at −80 °C. Electrophoresis was performed on a 9 % sodium dodecyl sulfate polyacrylamide-tris-glycine (SDS) gel, and the proteins were transferred to a nitrocellulose membrane (Bio-Rad Laboratories, Hercules, CA, USA). The membranes were then incubated with anti-DMP1 and anti-DSPP monoclonal antibodies (Santa Cruz) for 12 h at 4 °C. Next, the scaffolds were treated with horseradish peroxidase (HRP)-conjugated anti-mouse immunoglobulin G (IgG; Jackson ImmunoResearch, West Grove, PA, USA). The membranes were then exposed to an enhanced chemiluminescent substrate for HRP detection (Pierce, Rockford, IL, USA). Finally, immunoreactive proteins were visualized using the chemiluminescent substrate (SuperSignal West Pico), and the images were analyzed through densitometry with ImageJ software.

### 3.4. In vivo pulp capping model

To validate the effect of the bilayer scaffolds *in vivo*, the compact layer (PCL 600) was attached to aluminum foil, and the fibrillar layer of PCL/PEO was electrospun on top. Then, the bilayered scaffolds were cut into 1 × 1 mm specimens. As a result, the final scaffold consisted of two layers: a compact layer and a fibrillar layer. Next, these scaffolds were placed in a vacuum oven at RT for 24 h to evaporate any remaining solvent. The scaffolds were disinfected with UV light for 1 h on each side, followed by a 10-min wash with ethanol and two washes with PBS.

Twenty-four adult Fischer 344 rats (Envigo RMS Inc., Indianapolis, IN, USA), each weighing 250–300 g, were subjected to general anesthesia via intraperitoneal injections of ketamine (50 mg/kg; Hospira, USA) and xylazine (5 mg/kg; Akorn, USA). The experiments complied with the approved Institutional Animal Care and Use Committee (IACUC) protocol (PRO00010911). Pulp exposure was performed on the central part of the occlusal surface of the rats’ upper first molars using a sterilized #1/2 diamond bur (Kavo Kerr Group, Charlotte, NC, USA), and a #25 endodontic file confirmed the exposure (Fig. 10b–e). The rats were divided into three groups: MTA (White mineral trioxide aggregate Angelus; Angelus, Londrina, PR, Brazil), PCL/PEO, and PCL/PEO + CH. In the PCL/PEO and PCL/PEO + CH groups, bilayer scaffolds were cut with a 1-mm dermatological punch and applied to the pulp exposure site. The cavity was subsequently restored with conventional glass ionomer (Ketac Molar Easymix, 3 M ESPE, Saint Paul, MN, USA). One and four weeks post-treatment, the rats were euthanized by carbon dioxide inhalation. The right and left sides of the maxilla were then removed and preserved in a 10 % buffered formaldehyde solution.

#### 3.4.1. Morphological and chemical characterization of bilayer scaffold

After electrospinning, the bilayer scaffolds were stored under vacuum at RT for 72 h to ensure complete solvent evaporation. The bilayer scaffolds were cut with a sterile #15 scalpel and mounted on a 90° chamfer metallic stub, coated with gold and palladium (Au–Pd; 80–20), and the cross-section was analyzed using a field-emission scanning electron microscope (FEG/SEM, Tescan MIRA3 FEG-SEM, Tescan USA Inc., Warrendale, PA, USA). Additionally, because of CH incorporation in the fibrillar layer, the presence of calcium (Ca) was mapped using energy-dispersive spectroscopy (EDS).

#### 3.4.2. Micro-computed tomography (micro-CT)

To confirm biological functionality *in vivo*, micro-CT analysis was conducted on specimens collected after 7 and 30 days using the Scanco CT 100 system (Scanco Medical AG) to evaluate mineralized tissue formation and tissue mineral density within the pulp chamber beneath the applied material. Scans were taken with the following settings: 70 kV voltage, 114 μA current, and a voxel size of 12 μm, with an average exposure time of 500 ms per frame. After image reconstruction, the pulp chamber was manually outlined to define the region of interest (ROI). A consistent threshold for mineralized tissue volume deposition was applied across all samples (Sigma: 0.80; Lower Threshold: 320; Upper Threshold: 600) [72].

#### 3.4.3. Histological and immunohistochemical analyses

Following micro-CT analysis, the 7-day samples were demineralized in 10 % buffered EDTA and processed for histology. The samples were embedded in paraffin, and 5-μm-thick sections were stained with hematoxylin and eosin (H&E) and immunolabeled to assess inflammation and tissue neoformation within the pulp chamber. Images were captured at 10 × magnification using an ECHO Revolve microscope (BICO Company, San Diego, CA, USA). For cell quantification, color thresholding was applied using a low threshold of 10 and a high threshold of 130. ImageJ’s “Analyze Particles” function was used to count cells based on the following parameters: a circularity range minimum of 0.8 and a minimum particle size of 10 μm. Cell density was calculated as the number of cells per square millimeter by dividing the total cell count by the selected area. For immunofluorescence, tissue sections were prepared following the protocol described in a previous study [72]. Briefly, the following primary antibodies were used: anti-Nestin (19483-1-AP, Proteintech, Rosemont, IL, USA), anti-DMP1 (PA5-47621, Invitrogen), anti-CD31 (ab182981, Abcam, Cambridge, MA, USA), and anti-von Willebrand Factor (ab6994, Abcam), incubated at 4 °C for 24 h. A secondary antibody (Goat Anti-Mouse IgG H&L conjugated to Alexa Fluor^®^ 488, Abcam) was used for all primary antibodies. Sections were incubated with the secondary antibody for 2 h at RT in a light-protected environment. Cell nuclei were counterstained with DAPI using a Vectashield antifade mounting medium (Vector Laboratories, Newark, CA, USA). Fluorescence images were captured using the same ECHO Revolve microscope. Immunofluorescent labeling was quantified in ImageJ by separating the fluorescence channels and applying a threshold range of 100–255. The percentage of positively stained areas was calculated by measuring the fluorescence-expressing regions relative to the total tissue area [73,74]. All analyses were performed by a single and calibrated evaluator who was blinded to the experimental groups.

## 4. Statistical analysis

All data were assessed according to the assumptions of normality (Shapiro-Wilk) and homoscedasticity (Levene) to determine the appropriate statistical tests. Variables were analyzed using One- or Two-way ANOVA, followed by Tukey, Games-Howell, Sidak, or Dunnett’s post-tests. Calcium release and degradation comparisons were inferred with a 95 % confidence interval. All statistical inferences were based on the 5% significance level.

## Supplementary Material

Supplemental

## Data Availability

Data will be made available on request.

## Appendix A. Supplementary data

Supplementary data to this article can be found online at https://doi.org/10.1016/j.biomaterials.2025.123700.
