# Supplementary material for: Multifunctional bilayer scaffold for dental pulp protection and sustained calcium hydroxide release for mineralized tissue regeneration
Source: Biomaterials. Author manuscript; Available in PMC 2026 Mar 29. (PMC13033331; doi:10.1016/j.biomaterials.2025.123700)
Supplement: Supplemental [file NIHMS2154574-supplement-Supplemental.docx]

To determine the optimal calcium hydroxide (CH) concentration for incorporation into the PCL/PEO (50:50; v/v) polymer fibers, SEM images were obtained for the three highest concentrations that could be processed via electrospinning (**Fig. 1S**). Among these, the 0.2% (w/v) concentration was the highest that preserved a suitable fibrous morphology. Therefore, this concentration was chosen for the experiments described in section 3.3.


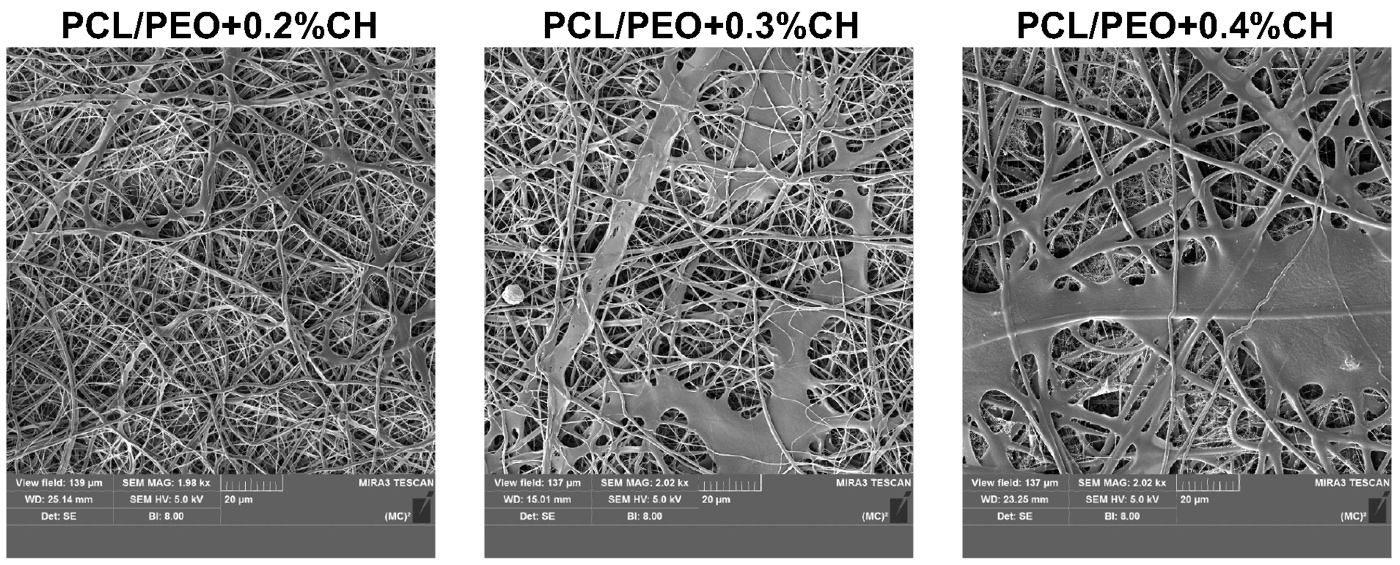


**Fig. 1S.** Scanning electron microscopy images (2000×) of the PCL/PEO fibrillar layer incorporated with different CH concentrations.
